# Determination of Parathion and Carbaryl Pesticides in Water and Food Samples Using a Self Assembled Monolayer/Acetylcholinesterase Electrochemical Biosensor

**DOI:** 10.3390/s8084600

**Published:** 2008-08-05

**Authors:** Valber A. Pedrosa, Josiane Caetano, Sergio A. S. Machado, Mauro Bertotti

**Affiliations:** 1 Instituto de Química, USP, São Paulo, SP, Brazil; 2 Instituto de Química de São Carlos, USP, São Carlos, SP, Brazil

**Keywords:** Biosensor, Pesticides, Acetylcholinesterase, Creek Water, Food

## Abstract

An acetylcholinesterase (AchE) based amperometric biosensor was developed by immobilisation of the enzyme onto a self assembled modified gold electrode. Cyclic voltammetric experiments performed with the SAM-AchE biosensor in phosphate buffer solutions (pH = 7.2) containing acetylthiocholine confirmed the formation of thiocholine and its electrochemical oxidation at E_p_ = 0.28 V vs Ag/AgCl. An indirect methodology involving the inhibition effect of parathion and carbaryl on the enzymatic reaction was developed and employed to measure both pesticides in spiked natural water and food samples without pre-treatment or pre-concentration steps. Values higher than 91-98.0% in recovery experiments indicated the feasibility of the proposed electroanalytical methodology to quantify both pesticides in water or food samples. HPLC measurements were also performed for comparison and confirmed the values measured amperometrically.

## 1. Introduction

The presence of pesticide residues and metabolites in food, water and soil currently represents one of the major issues for environmental chemistry. Pesticides are, in fact, among the most important environmental pollutants because of their increasing use in agriculture. The behavior of such polluting compounds in the environment is strongly determined by their physicochemical properties related to processes such as adsorption, leaching, vaporization and degradation [1,2]. Among the pesticides, organophosphate and carbamate compounds are the most widely used due to their high insecticidal activity and relatively low persistence [3]. These pesticides are toxic because they act as inhibitors of acetylcholinesterase , an enzyme that catalysis in a very efficient way the hydrolysis of the neurotransmitter acetylcholine. This enzyme is present in vertebrates and insects and its inhibition can disrupt the transmission of nerve impulses [4,5]. Therefore, the presence of residues of such pesticides in natural waters and in foodstuffs is of major concern for public health reasons.

The analysis of pesticides is usually carried out by gas and liquid chromatography with a selective-element detector [6,7]. Nevertheless, these procedures are expensive and frequently require laborious complex time-consuming sample treatment such as extraction of pesticides, extract cleaning, solvent substitution and clean-up steps. Furthermore, these approaches do not improve our understanding of the natural processes governing chemical species behavior, their transport, bioavailability, and their long-term impact on aquatic systems. The stability of samples during long-term storage is questionable, as they are subject to various biological, chemical and physical effects [8]. Finally, the analysis is usually performed in a specialized laboratory by skilled personnel and is not suitable for in situ application. These issues turn out to be a major problem when rapid and sensitive measurements are needed in order to take the necessary corrective actions in a timely approach. Accordingly, rapid, unfailing (sensitive and selective) and simple sensors for detecting pesticides continues to be an issue of interest in Electroanalytical research [9,10].

Biosensors for detection and quantification of pollutants have attracted extraordinary interest in recent years, because of the key role they play in the development of highly sensitive, selective chemical analysis, low cost and short analysis time associated with these devices. Biosensors based on the inhibition of acetylcholinesterase have been used for detection of pesticides in different samples [11-13] some different strategies being devised for enzyme immobilization. Self assembled monolayers (SAM) have been reported quite recently as another excellent choice to fabricate biosensors [14-20]. The properties of the SAM have been studied from electrochemical characterization by using cyclic voltammetry and impedance spectroscopy. The deposition of SAM does not inhibit the electron transfer process between the gold electrode and electrolyte [21]. Hence, in this paper a simple method were proposed for the determination of parathion and carbaryl in natural waters and foods samples using a SAM-AchE biosensor. The strategy is based on the action of some pesticides as potent irreversible inhibitors of cholinesterases, hence the extent of the inhibition effect can be analytically used to determine the pesticide concentration. The characteristic of the proposed electrochemical sensing system includes high sensitivity and selectivity, a wide linear range, minimal space and power requirements, and low-cost instrumentation. Accordingly, in this work we have demonstrated the analytical features of the proposed SAM-AchE biosensor for determination of parathion and carbaryl.

## 2. Results and Discussion

### 2.1. Water Analysis

**Figure 1.**
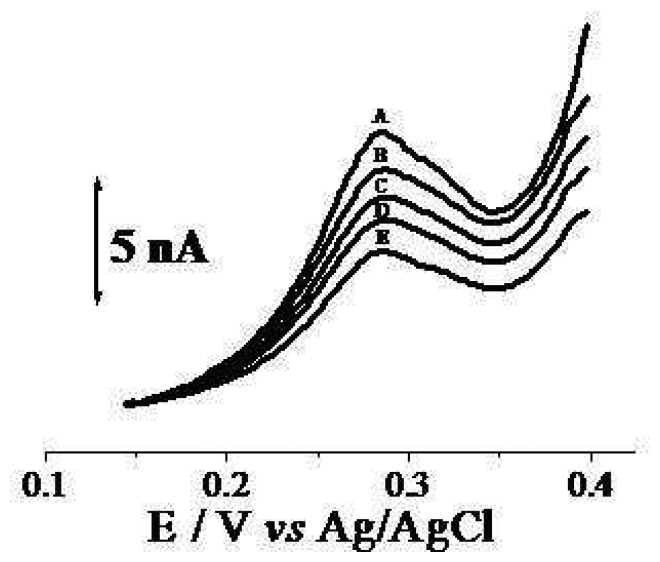
Cyclic voltammograms recorded with the biosensor in a 2.0×10^-3^ mol L^-1^ acetylthiocholine + 0.1 mol L^-1^ phosphate buffer solution pH 7.2 after addition of carbaryl in the different concentrations: 0 **(A)**; 1.0 **(B)**; 2.0 **(C)**; 3.0 **(D)** and 4.0×10^-6^ mol l^-1^**(E)**, scan rate = 50 mV s^-1^.

Environmental monitoring and food control generally require the analysis of a large number of samples and there is a need for low, rapid and sensitive methods of analysis. The choice of cholinesterase as a biorecognition element enables the simultaneous detection of a wide group of related toxic compounds such as organophosphorus and carbamate pesticides. The enzymatically catalysed hydrolysis of acetylthiocholine was evaluated by using the fabricated SAM-AchE biosensor by cyclic voltammetry in 0.1 mol L^-1^ phosphate buffer solution (PBS) at pH = 7.2. At these experimental conditions thiocholine is produced and its anodic oxidation gives rise to a peak at 0.28 V (peak 1) as shown in Figure 1. This relatively low working potential needed for the anodic process is advantageous because it prevents the oxidation of possible interfering species existing in the samples to occur. Figure 1 shows a clear demonstration of the inhibition effect of a pesticide (carbaryl) on the biosensor response as current decreases with increasing the concentration of the pesticide. This peak was used to follow the inhibition of enzymatic reaction by pesticides and therefore this potential was selected as the working potential.

**Figure 2.**
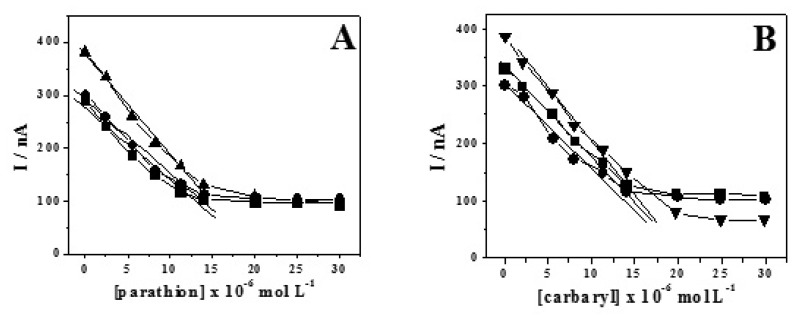
Analytical curves obtained by amperometry (E = 0.28 V) for parathion **(A)** and carbaryl **(B)** in electrolytes prepared with different pure (▲) and contaminated water samples point 1 (●) and point 2 (■).

The pesticides were determined in spiked natural water samples without any previous extraction, clean-up or pre-concentration steps. The matrix effect of water samples collected from urban creeks (Gregorio and Monjolinho creeks, São Carlos County, Brazil) was evaluated as these natural samples were directly used to prepare the supporting electrolyte solution. Calibration curves were obtained for both pesticides with concentrations varying from 2.0 to 30.0×10^-6^ mol L^-1^ in 0.1 mol L^-1^ PBS supporting electrolyte, pH 7.2 (Figure 2). The DL obtained by using equation 1 was found to be in the range 9.0-10.3 μg L^-1^, which is close to the concentration limit of 10 μg L^-1^ carbaryl and parathion recommended by the Environment Protection Agency (EPA) [22] as the maximum contaminant level for waste waters. Natural water samples used in this work did not contain detectable amount of both pesticides, hence they were spiked with parathion and carbaryl. In such artificially contaminated samples, recovery experiments were performed by the standard addition technique. An initial Parathion concentration of 6.0×10^-6^ mol L^-1^ was added to each sample. In the sequence, several standard solutions were added, altering the concentration in the amperometric cell up to 10×10^-6^ mol L^-1^ These results are presented in Table 1, which shows that recovery values around 94.0-96.0% were found for the polluted waters. The small matrix influence on the analytical sensitivity has frequently been observed and related to the organic matter dissolved in natural waters, mainly humic and fulvic acids [23]. All values reported reflect small loses in the procedure, thus indicating the suitability of method proposed for analytical measurements.

**Figure 3.**
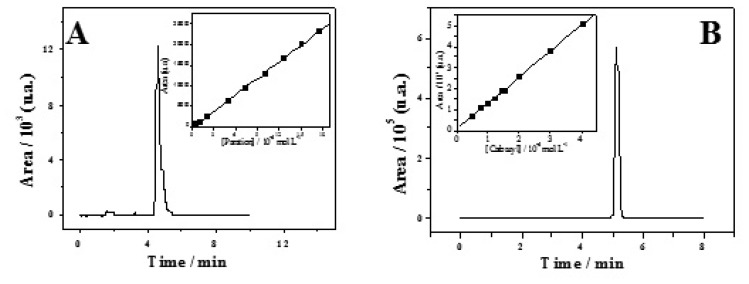
Chromatograms of parathion **(A)** and carbaryl **(B)** standard solutions (5.0×10^-5^ mol l^-1^ in 0.1 mol l^-1^ phosphate buffer, pH=7.2). Insets correspond to calibration plots for both pesticides.

Figure 3 shows chromatograms for parathion and carbaryl in 0.1 mol L^-1^ PBS electrolyte (pH=7.2), well-defined peaks appearing at retention times of 4.5 and 5.4 min. respectively. In the same figure analytical curves for both pesticides are shown in the concentration interval 2.0 to 30.0×10^-6^ mol l^-1^. Water samples were analyzed by HPLC in order to validate the results obtained with the biosensor. The water samples were filtered before HPLC analysis only to avoid clogging problems and then spiked with the minimum detectable amount of parathion and carbaryl. The corresponding analytical parameters are presented in Table 1 together with that obtained in pure water, for comparison. As it can be observed from the slopes of the analytical curves, the determination of parathion and carbaryl in contaminated waters has a lower sensitivity when compared to pure water. Moreover, recovery experiments were carried out for the quantification of parathion and carbaryl in the different water samples following the procedure presented in the experimental section. For the samples collected at points 1 and 2 values around 92-95 % for both pesticides were found, respectively (Table 1).

### 2.2. Fruit sample testing

Organophosphate and carbamate are some of the most widely used insecticides in citrus fruit, tomato and apple cultures. Due to its permitted use in such cultures, the methodology proposed was applied in order to evaluate the occurrence of matrix effects in the electroanalytical determination of parathion and carbaryl residues directly in the samples, without pre-treatment or clean up steps. The suitable performance of the electrochemical biosensor was investigated by analyzing several food samples to which different amounts of pesticides were added to give concentrations in the range between 6.0×10^-6^ and 5.0×10^-5^ mol L^-1^. The inhibition of AchE caused by the incubation in the spiked food was compared with the inhibition observed when equivalent pesticide concentrations were present in the buffer solution. The experiments were carried out in triplicate and pesticide concentrations were determined using the Equation 2. The extract of the juices was prepared according to procedures described in the experimental section and had their pH values adjusted to 7.2 with appropriate volumes of NaOH solution. 20 mL aliquots of each sample were added to the electrochemical cell for the recovery experiments. The concentration of pesticides in the extract was determined by amperometry using the standard addition method and the results obtained are presented in Table 2.

In order to validate the previous electroanalytical results, HPLC quantification experiments were carried out using 20 μL aliquots of the appropriate extract obtained from the different samples (see experimental section). The experiments were carried out in triplicate and the pesticides recovery concentration was determined using the Equation 2 (Table 2). The obtained values ranged from 77-90 % and were slightly higher than those obtained with the biosensor conditions (see Table 2), suggesting that the biosensor has the same better efficiency as compared with HPLC in fruits analysis.

Results of parathion and carbaryl determinations in water and food samples by using the proposed biosensor and HPLC were in close agreement. The major advantage of the biosensor is that it allows the analysis of a large number of samples with no need of clean-up steps such those required in chromatographic methods. Frequent routine analyses can be safely carried out using this simpler and less expensive electroanalytical method without losses in either reliability or precision.

## 3. Conclusion

A pesticide biosensor was successfully developed and the obtained results demonstrate that its use as an integrated acetylchlolinesterase self assembled amperometric device accomplishes the requirements of precision, speed, sensitivity, simplicity and low cost. This proposed analytical tool is suitable to be employed for rapid judging of the quality of fruit and natural waters. The concept involved in the fabrication of the SAM-AchE biosensor can be extended to assemble other biological molecules, such as antiboby, antigen, and DNA, to the CNT surface for wide bioassay applications.

## 4. Experimental Section

### 4.1 Materials

3-Mercaptopropionic (3-MPA) (Aldrich 99%), acetylcholinesterase (AchE) E.C. 3.1.1.7 Type XII-S from bovine erythrocytes 1.0 U/mg, acetylthiocholine iodide (ACh) (99% purity), glutaraldehyde (grade II, 25% aqueous solution) and N'cyclohexy-N′(2-morpholinoethyl)carbodiimide methyl-p-toluenesulfonate (EDC) (99%) were obtained from Sigma-Aldrich. Parathion and carbaryl standards were obtained from Sigma-Aldrich. All the solutions were prepared by dissolving the compounds or diluting concentrated solutions in deionized water processed through a water purification system (Nanopure Infinity, Barnstead).

### 4.2 Apparatus

The electrochemical cell was constituted of a gold disc electrode (geometric area = 10 mm^2^) as working electrode, a platinum wire as auxiliary and a Ag/AgCl electrode (3.0 mol L^-1^ KCl solution) as reference. Cyclic voltammetric and amperometric experiments were carried out using a 273 EG&G PARC instrument. The biosensor was prepared by using a procedure described elsewhere [24]. AChE was immobilized onto the SAM electrode by cross-linking with glutaraldehyde. This was accomplished by adding 20 μL (11.8 U mg) of AChE solution (prepared in phosphate buffer solution, pH = 7.2) to the electrode surface and leaving for 20 minutes to form a wet film. The electrode was then treated with concentration glutaraldehyde 2.5% v/v solution during 1 minute. The biosensor was kept in dry conditions at 4° C. Acetylthiocholine iodide (Ach) was used as the AChE substrate in all experiments.

Quantitative determinations of parathion and carbaryl were also carried out by HPLC-UV technique. A model. SCL-10AVP Shimadzu system equipped with a model, LC-10ATVP pumping unit and a spectrophotometric UV/Vis detector (SPD-10AVP) were used in these experiments. Data were processed on a Shimadzu LC Work-Station Class LC-10. The HPLC conditions were: a Li-Chrosorb RP-18 column (250 mm, 4.6 mm, 5 mm, Merck), with a RP-18 pre-column (30 mm, 4 mm, 5 mm, Merck); the mobile phase was 70/30 v/v acetonitrile/water with 1% v/v acetic acid, at a flow-rate of 1.0 ml min^-1^. The injection volume was 20 μL and detection was performed at a wavelength of 270 nm for parathion and 220 nm for carbaryl. The standard addition method was used for analytical determinations in real samples.

### 4.3 Water Sample collection

The water samples were evaluated by analyzing natural water samples collected in two local creeks at different points in the São Carlos city, namely, before (point 1, Monjolinho) and after crossing the town (point 2, Gregorio). Water samples were collected in glass bottles during the dry season and kept under refrigeration (4° C) for no longer than one week. The amount of organic matter in the water samples increases between point 1 and point 2, as indicated by biological (BOD) and chemical oxygen demand (COD) values: 6.0 and 12.0 mg L^-1^ (BOD) and 19.0 and 33.0 mg L^-1^ (COD), respectively. The electrolytes were prepared by dissolving the salts necessary for the phosphate buffer in either pure or natural waters and the measurements were performed without pretreatment of the solutions, but the pH was properly adjusted to the desired value in each case. As the analyzed samples did not contain both pesticides at detectable concentrations, recovery experiments were performed by spiking the samples with known amounts of each pesticide, followed by standard additions. All measurements were performed in triplicate.

The standard deviation of the mean current values (*S_B_*) measured at the each pesticide reduction potential for ten voltammograms of the blank solution in electrolytes prepared with different water samples was used to determine the detection limit (DL) values accordingly to the IUPAC definitions, these are related by Equation (1) [25].

(1) $$\text{DL}=\frac{3{\text{S}}_{\text{B}}}{\text{b}}$$

where b is the slope of the analytical curve.

All measurements were performed in triplicate. The recovery efficiencies (R%) for the different systems under investigation were calculated using Equation (2), where the value [Pesticide] found refers to the concentration obtained by extrapolation of the analytical curve in the corresponding spiked water samples

(2) $$\%R=100\;\frac{[\text{pesticide}]\;\text{found}}{[\text{pesticide}]\;\text{added}}$$

The precision and accuracy of methodologies were tested with different standard solutions of each pesticide and the relative standard deviations (RSD) were calculated using the Equation (3):

(3) $$\text{RSD}=\frac{Sb}{x}$$

where *S*b is standard deviation of the mean current values obtained and *X* is the mean peak current value.

### 4.4. Extraction step in fruits

#### Sample Treatment for Electrochemical Measurements in Fruits

40 g of tomato and apple samples were directly transferred to an electrochemical cell after triturated and spiked with different amounts of both pesticides. Then, the mixture was mechanically stirred and electrochemical measurements were performed under the conditions described in the Experimental Section. Orange juices were extracted from their respective fruits and 20 mL of the juice were transferred to an electrochemical cell and artificially contaminated with known amounts of both pesticides.

#### Extraction for Matrix Solid-Phase Dispersion (MSPD) analysis

Untreated fruit samples were processed as received in their unprocessed and unwashed form and kept in a freezer. After the sample treatment previously described they were spiked with different amounts of both pesticides and introduced into a 15 mL cell containing 3.0 g of anhydrous sodium sulfate placed over 0.5 g of silica gel. The column was prepared in the laboratory and conditioned with 10 mL of ethyl acetate. A 10 mL round-bottomed flask was positioned below the column to collect the eluate. The eluate was then concentrated using a rotary vacuum evaporator (40-45 °C water bath, reduced pressure), and the final volume was adjusted to 1.0 mL. A 20 μL portion of the extract was analyzed by HPLC.

## Figures and Tables

**Table 1.** Analytical parameters for parathion and carbaryl determinations in pure and contaminated water samples by using amperometry with the SAM-AchE biosensor and HPLC.

	***Pesticide***	***Sample***	***r***	***Sb (μA)***	***b (A/mol L****^-1^****)***	***DL (μg L****^-1^****)***	***Recovery (%)***
*SAM-AchE*	Paration	Pure	0.994	0.017	1.9×10^-4^	9.3	-
		Point 1	0.989	0.014	1.8×10^-4^	10.3	96 ± 2
		Point 2	0.980	0.013	1.5×10^-4^	9.7	94 ± 1

	Carbaryl	Pure	0.970	0.015	2.6×10^-4^	9.0	-
		Point 1	0.992	0.014	1.6×10^-4^	9.6	95 ± 1
		Point 2	0.990	0.013	1.7×10^-4^	9.8	94 ± 2

HPLC*	Parathion	Pure	0.999	16.85	1.3×10^10^	1.1	-
		Point 1	0.997	14.30	0.2×10^10^	5.1	95 ± 3
		Point 2	0.994	13.90	0.1×10^10^	6.4	93 ± 2

	Carbaryl	Pure	0.999	8.80	0.2×10^10^	3.1	-
		Point 1	0.998	7.90	8.0×10^9^	8.3	94 ± 2
		Point 2	0.993	7.80	8.3×10^9^	9.0	92 ± 1

**Table 2.** Recovery values for parathion and carbaryl in fruit samples.

***Sensor***	***Sample***	***Pesticide***	***Concentratio n added***	***Concentratio n found***	***Recovery (%)***
		Parathion	6.0×10^-6^	5.7×10^-6^	95 ± 2

			5.0×10^-5^	4.6×10^-5^	92 ± 3

***SAM-AchE***	Tomato	Carbaryl	6.0×10^-6^	5.5×10^-6^	92 ± 3

			5.0×10^-5^	4.7×10^-5^	94 ± 3

		Parathion	6.0×10^-6^	5.2×10^-6^	87 ± 2

			5.0×10^-5^	4.4×10^-5^	8.8 ± 1

***HPLC***	Tomato	Carbaryl	6.0×10^-6^	4.7×10^-6^	78 ± 2

			5.0×10^-5^	4.2×10^-5^	84 ± 2

		Parathion	6.0×10^-6^	6.1×10^-6^	101 ± 3

			5.0×10^-5^	4.9×10^-5^	98 ± 3

***SAM-AchE***	Apple	Carbaryl	6.0×10^-6^	5.8×10^-6^	97 ± 2

			5.0×10^-5^	4.7×10^-5^	94 ± 2

		Parathion	6.0×10^-6^	5.4×10^-6^	90 ± 1

			5.0×10^-5^	4.4×10^-5^	90 ± 2

***HPLC***	Apple	Carbaryl	6.0×10^-6^	4.6×10^-6^	77 ± 2

			5.0×10^-5^	4.3×10^-5^	82 ± 3

		Parathion	6.0×10^-6^	5.8×10^-6^	97 ± 3

			5.0×10^-5^	4.9×10^-5^	98 ± 2

***SAM-AchE***	Orange	Carbaryl	6.0×10^-6^	5.6×10^-6^	93 ±2

			5.0×10^-5^	4.8×10^-5^	96 ± 2
